# Surface Coatings of Reinforcement Phases in Magnesium Matrix Composites: A Review

**DOI:** 10.3390/ma16247560

**Published:** 2023-12-08

**Authors:** Shiyi Wu, Bin Chen

**Affiliations:** School of Materials Science and Engineering, Shanghai Jiao Tong University, Shanghai 200240, China; wushiyi@sjtu.edu.cn

**Keywords:** magnesium matrix composites, coating treatment, reinforcements, interface

## Abstract

Magnesium matrix composites have been extensively investigated due to their light weight and machinability. The interfaces are the most important part of these composites, and their properties determine the properties of composites to a large extent. However, there are still many problems with interface bonding. The reinforcements are faced with the dilemma of poor dispersion, bad interfacial reaction, and poor wettability, which limits further improvements in the mechanical properties. Surface coating treatment of reinforcements is considered to be one of the effective methods to protect reinforcements and modify the interface. This review presents an overview of different coating materials on various reinforcements. The major roles of coatings in the composites and the properties of the composites are discussed. Future directions and potential research areas in the field of magnesium matrix composites reinforced with coated reinforcements are also highlighted.

## 1. Introduction

With the gradual improvement in energy conservation and environmental protection requirements, lightweight materials are receiving increasing attention. As the lightest structural materials, magnesium (Mg) alloys have been extensively studied in recent years. Because of their superior properties, such as castability and machinability, Mg-based materials have been widely employed in the aerospace, automobile, biomedicine, and other industries [1,2,3,4]. However, there are still many limitations, such as low ductility, low elastic modulus, and poor corrosion resistance, which hinder the broad application of Mg alloys [5,6,7]. In order to improve the mechanical properties of Mg alloys, there are usually two methods used: the addition of alloying elements [8,9,10] or the introduction of reinforcements [11]. In particular, due to the various excellent properties brought by the rich variety of reinforcements, Magnesium matrix composites (MMCs) have been extensively investigated [5,12]. 

Many reinforcements can be adopted in MMCs, such as carbon materials (carbon fiber (CF) [12], carbon nanotubes (CNT) [4], and graphene [13], etc.), ceramic materials (SiC [14], Al_2_O_3_ [15], AlN [16], B_4_C [17], etc.), and metals (Ni [18], Ti [19], Nb [20], etc.). Classified from the dimension of reinforcements, particles [14], whiskers [21], fibers [22], and 2D fibers [23] are utilized in composite systems. MMCs strengthened with these different reinforcements all have good specific strength, modulus, and hardness values. The particle-reinforcing MMCs have the characteristics of easy production, low cost, and isotropy [2,24]. The addition of these reinforcements improves the performance of MMCs through effective load transfer, grain refinement, induced crack deflection, and obstruction of dislocation. Furthermore, load transfer is the main strengthening mechanism of MMCs, and the interface is the “link” connecting the Mg matrix and reinforcements [25,26]. The properties of the interfaces determine the mechanical, damping, high temperature, and corrosion resistance properties of MMCs to a large extent [27,28,29]. However, there are still many problems with the interface bonding. Except for metal reinforcements, the reinforcement phases, especially nano-sized particles, are faced with the dilemma of poor dispersion, which limits the further improvements in the mechanical properties in MMCs [21]. In addition, the properties of MMCs are usually affected by poor interface wettability, weak bonding strength, and lousy interface reaction [30,31]. Therefore, optimizing the interface between reinforcements and the Mg matrix is the key to improving the performance of MMCs. 

**Figure 1:**
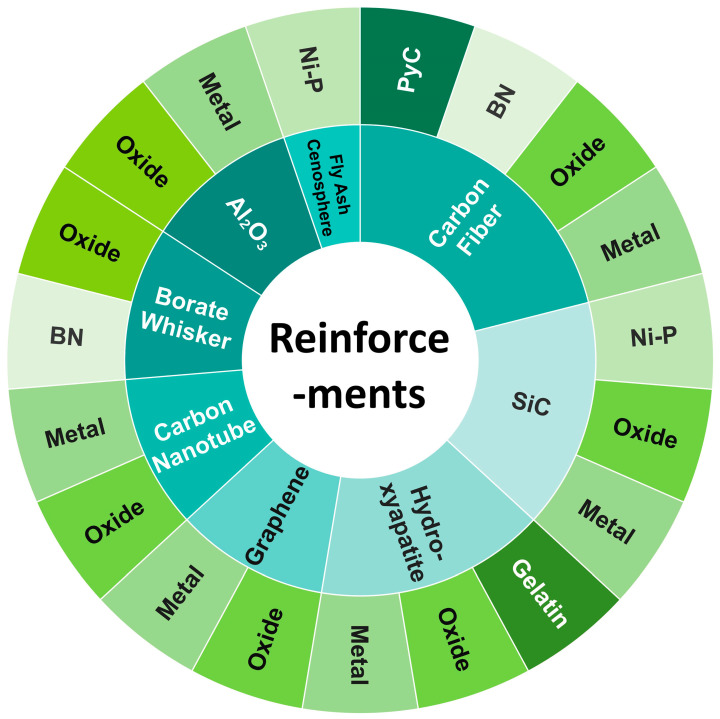
The reinforcements applied in MMCs and coatings adopted on the reinforcements.

Many Mg devices are often coated with alloy or composite coatings to improve wear and corrosion resistance [32,33,34]. Similarly, the surface coating treatment of reinforcements is considered to be one of the effective methods to protect reinforcements and modify the interface. Generally, coating treatments include chemical vapor deposition (CVD) [35], electroless plating [36], sol–gel [22], etc. The presence of coatings can improve the dispersion and the interfacial wettability with the Mg matrix. Furthermore, the processing of MMCs frequently necessitates the application of elevated temperatures and pressures. In this regard, the implementation of coatings serves to protect the reinforcements against potential harm throughout the processing procedure. A variety of materials are used for coating, including metals, oxides, ceramics, etc. By summarizing the current research, the main reinforcements and corresponding coating materials used in MMCs are shown in Figure 1. 

In this review, we address the main research results on the surface coating treatment of reinforcements in MMCs. According to the types of reinforcement, different coating treatments and their properties are summarized. The interface between the coating and matrix and the interface between the coating and reinforcement are discussed. Moreover, the effects of different coatings and their influence on the MMCs’ properties are also compared and analyzed.

## 2. Carbon-Reinforced MMCs

Carbon materials are one of the most widely used and the most frequently coated reinforcements. By modifying the combination of carbon atoms, numerous different structures of different scales can be obtained [37,38,39]. The flexibility of modifying the physical properties and their availability have attracted much attention to carbon materials. The carbon reinforcements used in MMC include CF, CNT, graphene, and so on. Their application may be hindered by problems such as poor wettability, agglomeration, and thermal decomposition [40,41]. The following sections review the coating treatments for different carbon reinforcements in carbon-reinforced MMCs.

### 2.1. Carbon Fiber

**Figure 2:**
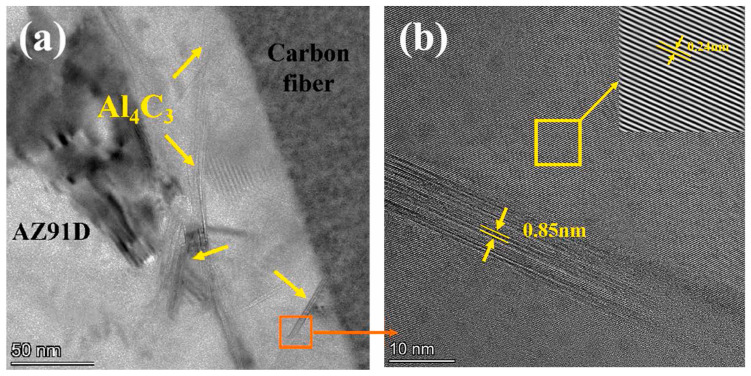
Typical TEM images of CF/AZ91D composite: (**a**) microstructure of interface; (**b**) HRTEM image of orange solid line box in (**a**) (inset on the top right is the IFFT image of solid line box in (**b**)). Reproduced from [22] with permission of the publisher (Elsevier).

CF is an ideal reinforcement for MMCs because of its excellent properties, including high tensile strength, low density, and low thermal expansion [42,43]. However, due to the difference in interfacial characteristics of the CF and Mg matrix, the direct application of CF is restricted. Furthermore, interfacial reactions exist in the CF/Mg-Al system, and the formation of plate-shaped Al_2_MgC_2_ or Al_4_C_3_ shown in Figure 2 strongly influences the bonding strength [44]. Two methods are usually used to enhance the interface adhesion: surface oxidation and coating treatment [45]. However, the damage to the CF caused by oxidation treatment often reduces its mechanical properties, which can even reach more than 45% after 3 h of acid treatment [46,47]. Therefore, coating treatment is a simple and effective method that has attracted the attention of researchers. In addition to the general role mentioned above, coatings are particularly important for CF reinforcements to improve interfacial load transfer [48]. Coating materials applied for CF reinforcements in MMCs include metals, oxides, pyrolytic carbon (PyC), Ni-P, TiN, and BN. The research status of different types of coatings is introduced below. 

#### 2.1.1. Oxide Coatings

**Figure 3:**
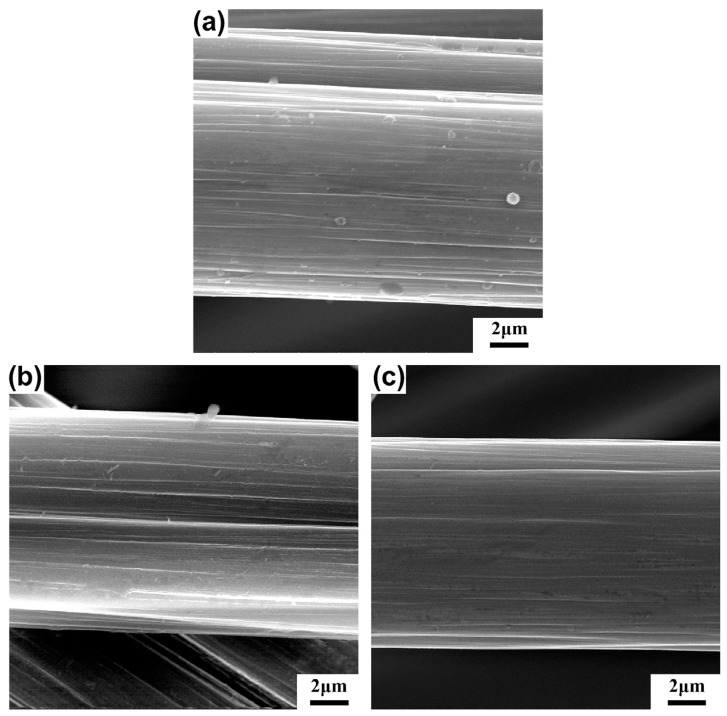
SEM images of (**a**) unsized, (**b**) Al_2_O_3_, and (**c**) TiO_2_ sol-coated CF after calcination at 735 °C for 60 min, respectively. Reproduced from [49] with permission of the publisher (Elsevier).

Various oxide coatings prepared by sol–gel methods have been investigated to improve the interface of CF-reinforced MMCs, including SiO_2_, Al_2_O_3_, TiO_2_, and ZrO_2_. As shown in Figure 3, through the sol–gel method, uniform oxide coatings with neither crinkle nor crack can be obtained on the CF surfaces [49,50]. In addition, Li et al. [45] proposed that the interfacial shear strength and fracture toughness of single TiO_2_-coated CF witnessed a significant improvement due to high-temperature curing. 

Oxide coatings can react with the matrix to form nano-scale MgO, which promotes wettability. The interfacial reactions obey the following equations [22,49,51,52,53]:

(1) $$SiO_{2}+2Mg\to 2MgO+Si$$

(2) $$Al_{2}O_{3}+3Mg\to 3MgO+2Al$$

(3) $$TiO_{2}+2Mg\to 2MgO+Ti$$

(4) $$ZrO_{2}+2Mg\to 2MgO+Zr$$

The existence of MgO can prevent bad interfacial reactions and inhibit element diffusion, which protects CFs from damage and keeps their mechanical properties. In ZrO_2_-CF/AZ91D composites, the formation of the double-layer interface (ZrO_2_-MgO) plays this role [22]. MgO preferentially forms on the surface of ZrO_2_, and the double-layer interface impedes the diffusion of C and Al elements, which prohibit the formation of Al_4_C_3_. Another critical influence of ZrO_2_ interfacial layers is effective load transfer and high energy absorption, which both improve the strength and toughness of composites [22,51,53].

However, the content of alloying elements in the Mg matrix can affect the effect of the coating. For example, the effectiveness of SiO_2_ coatings in an CF/Mg-Al system is relevant to the Al content in the matrix. The SiO_2_-CF/Mg-1Al composite provides the best mechanical properties. When the Al content in the matrix is bigger than 1 wt%, SiO_2_ coatings are fully depleted, and extensive carbides are formed in the interface, which leads to brittle fracture behavior and a lower tensile strength of the composites [50,54,55]. On the contrary, the interfaces between TiO_2_ coatings and matrix are not affected by the Al content, and the strength decrease is attributed to the increase in Mg_17_Al_12_ precipitates [56]. In addition, the volume expansion caused by the interface reaction will also affect the effect of the coating. The volume expansion rate of Al_2_O_3_ coatings reaches 21.8%, which causes the formation of crystal defects, and the coating layers react entirely [49]. These phenomena eventually result in a decrease in their strengthening and protection effect. Unlike Al_2_O_3_ coatings, the product Ti of the interfacial reaction of TiO_2_ is difficult to dissolve into the matrix, and Ti will accumulate near the interface, which inhibits the diffusion and further reaction [49]. According to the above discussion and the conclusion, the strengthening ratios of CF/Mg do not depend on the volume fraction of CF but on the interfacial microstructure. TiO_2_ and ZrO_2_ are a better coating material choice than SiO_2_ and Al_2_O_3_ [53]. In addition, Cho et al. [57] prepared hybrid reinforced MMCs using an in situ interfacial reaction of TiO_2_-coated carbon nanofiber, which dramatically improved the AZ91 matrix. 

#### 2.1.2. Pyrolytic Carbon Coatings

PyC is another coating material frequently used to improve interfacial wettability and tailor load transfer, and it has been used since 2002 [58]. PyC coating is deposited on CF through the CVD method. Li et al. [30] proposed a liquid-solid extrusion and vacuum pressure infiltration technique (LSEVI) to fabricate MMCs reinforced with PyC-coated CF. The technique could reduce the temperature and pressure during fabrication, which resulted in the degradation of carbon.

**Figure 4:**
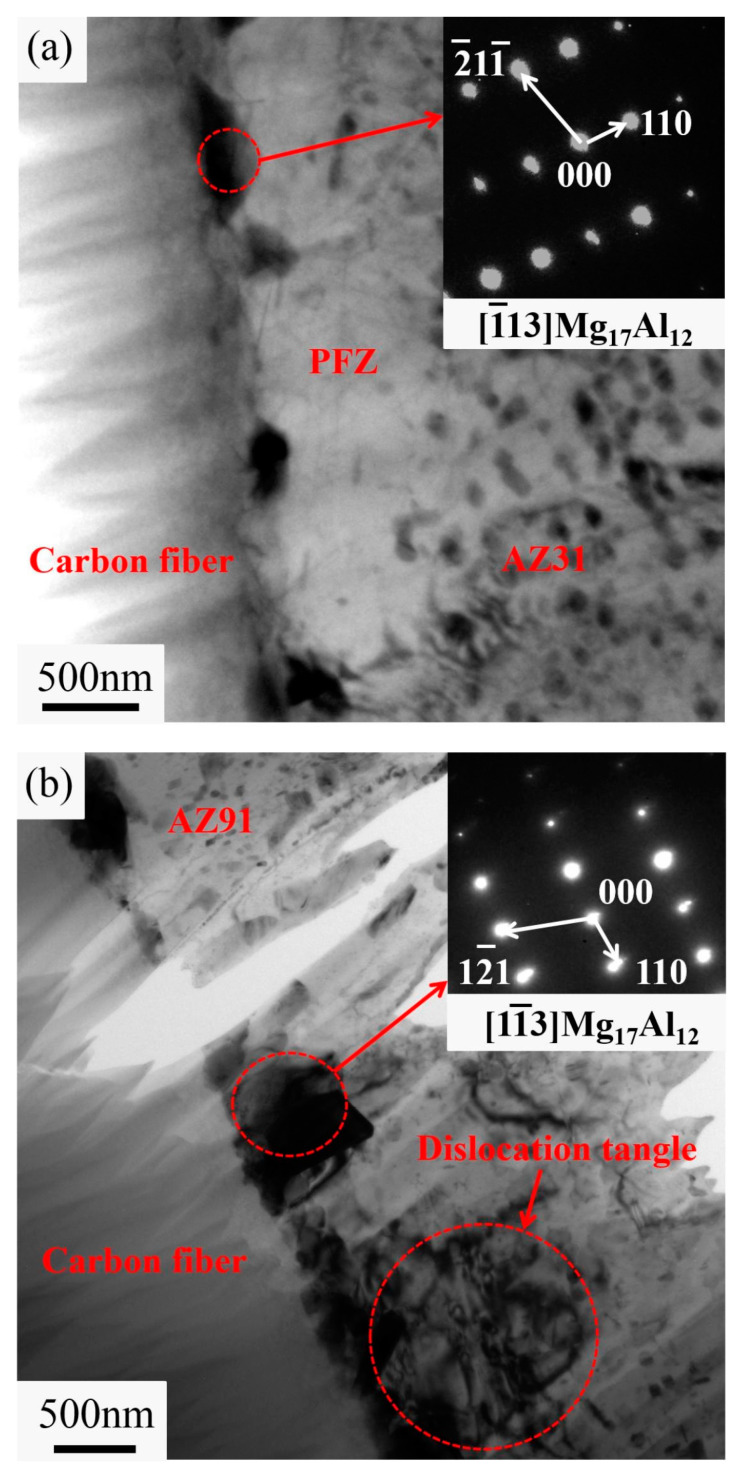
TEM images of the composites. (**a**) PyC-CF/AZ31 composite; (**b**) PyC-CF/AZ91 composite. Reproduced from [35] with permission of the publisher (Elsevier).

The interface of PyC-coated CF is shown in Figure 4. PyC coatings prevent interfacial reactions, and only some block-shaped precipitates are observed at the interface [23,35,59]. The fine and uniform precipitates and the nanocrystalline formed in the Mg matrix are beneficial to enhancing the MMCs. The coarse interfaces of the PyC adjust the stress on the interfaces and provide the desired bonding strength, which obviously improves the mechanical properties by more than 80% [23,60,61]. In addition, PyC coatings could improve the damping performance of AZ91-based MMCs between room temperature and 170 degrees [62]. Li et al. [63] proposed that compared to TiO_2_ coatings, PyC coatings offered a more significant improvement in the properties with a higher cost. Therefore, the choice of oxide coatings or PyC coatings needs to be made according to the actual conditions. 

#### 2.1.3. Metal Coatings

**Figure 5:**
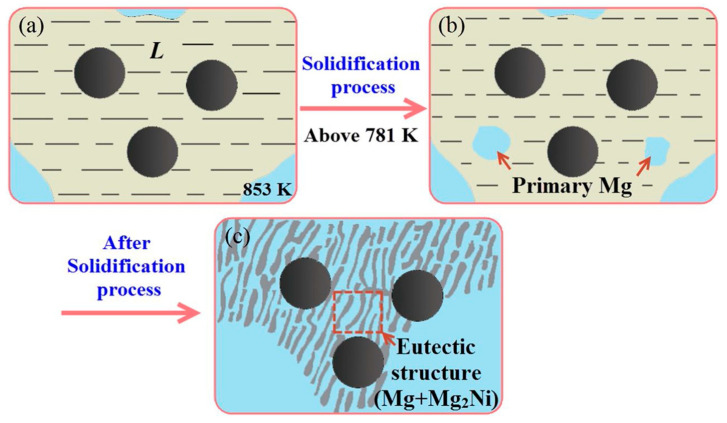
Schematic diagram of the microstructure of CF/Mg composites fabricated at 853 K: (**a**) during the fabrication process at 853 K; (**b**) during the solidification process above 781 K; (**c**) after the solidification process. Reproduced from [65] with permission of the publisher (Elsevier).

Compared to oxide coatings prepared with the sol–gel method, metal coatings prepared with electroless plating are cheaper, simpler, and more universal. The electroless approach is much simpler than CVD and electrodeposition [64]. Ma et al. [36] conducted in-depth research on the Cu electroless plating process and the growth mechanism of the Cu coating. The pretreatment of CF can promote the formation of metal coatings. The other most used metal coating element is Ni. Ni diffuses into the Mg matrix, and Mg_2_Ni compounds are formed. The corresponding evolution process is shown in Figure 5. A fine Mg_2_Ni structure provides better interfacial wettability, combination, and effective load transfer, but coarse Mg_2_Ni leads to rapid interfacial failure [65,66,67]. Some studies have also shown that the strengthening mechanism also includes dislocation strengthening caused by a thermal expansion coefficient mismatch, and CF can inhibit dislocation slip [68]. In a Mg-Al-Ca system, the hardness improvement is attributed to grain refinement, Al_3_Ni formation, and vapor-grown CF reinforcement [69].

A small number of studies have also used electroplating methods. Peng et al. [70] synthesized Zn-coated CF, and the MMC had better bonding properties than that of an MMC with SiO_2_-coated CF. 

#### 2.1.4. Other Coatings

In addition to the above coating materials, several coatings prepared using the CVD method have also been investigated. Reischer et al. [71] studied hexagonal BN coatings with a similar nanostructure to PyC, and the bending strength was improved by 42% up to 1620 MPa. Homma et al. [72] proposed Si-coated carbon nanofibers, and the coating led to better wettability with an AZ91D matrix. In addition, TiN has also been applied as a coating material of CF and has improved the properties of MMCs to a certain extent [48].

### 2.2. Carbon Nanotubes

CNTs are also considered an ideal enhancer due to their high strength, Young’s modulus, and thermal conductivity [73]. Well-dispersed CNTs can dramatically enhance MMCs. CNTs can bridge incipient cracks and inhibit the dislocation motion, which increases the fracture strength and toughness of MMCs [74,75]. However, the wettability of Mg and sp^2^ bonded carbon is relatively low, indicating that CNTs will agglomerate in molten Mg [76]. Many coating materials, including metals and oxides, have been investigated to solve these problems. 

The most commonly used metal coating is Ni, and common preparation methods include electroless plating and CVD. The mechanism of the Ni-enhancing interface bonding is an in situ reaction that generates Mg_2_Ni [77]. Both continuous coatings and nanoparticle coatings can improve interface wettability, which increases the content of CNTs that can be dispersed uniformly [78,79]. Additionally, coatings can potentially break the van der Waals forces among the CNTs, which prevents them from re-agglomerating [80]. Moreover, the content of uniformly dispersed Ni-coated CNTs varies in these studies owing to the differences in the preparation methods of MMCs [31]. But once the content exceeds the optimal content, the agglomeration will decrease the interfacial load transfer efficiency [81]. The grain refinement brought about by the addition of reinforcements increases the strength while the ductility is not affected [78,79]. In addition, Ding et al. [82] balanced the relationship between strength and plasticity by introducing large-sized AZ61 particles as soft phases to MMCs reinforced with Ni-coated CNTs. Zhou et al. [83,84,85] investigated the strengthening mechanisms and mechanical properties of Mg reinforced with Ni-coated CNTs by molecular dynamics simulations. They demonstrated that Ni coatings drastically increased the interfacial bonding and provided effective load transfer. Appropriately increasing the coating thickness can further improve the strengthening effect [85]. For multi-walled CNTs, the increase in the wall number and inner diameter results in interface improvement [83]. In addition to Ni, some studies have used Pt [80], Cu [86], and Al [87] as coating materials, which have also improved the strengthening effect of CNTs.

**Figure 6:**
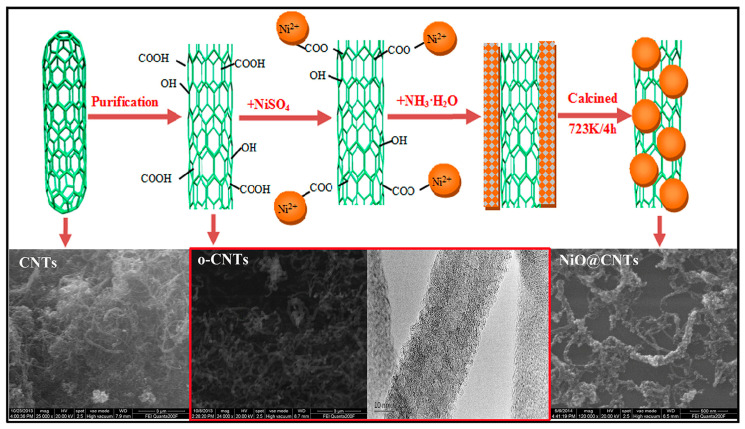
Schematic illustration of preparation of NiO-coated CNTs. Reproduced from [94] with permission of the publisher (Elsevier).

The preparation methods of oxide coatings mainly contain three types: the hydrothermal method [88], the one-step precipitation process in the Mg/CO system [89], and the chemical method shown in Figure 6. Qiu et al. [90] demonstrated that the interfacial bonding and stability are improved by MgO coating treatment through experiments and first-principles calculation. Different oxide coatings, including TiO_2_ [91], MgO [92,93], and NiO [94], can enhance the interfaces between CNTs and the alloy matrix. Nano-scale contact and diffused bonding are formed at the interfaces between CNTs and MgO, and semi-coherent interfaces are formed between MgO and α-Mg. An interfacial reaction to generate MgO and Mg_2_Ni is observed between NiO coatings and the Mg matrix. These fine interface combinations improve the mechanical properties of MMCs. In addition, introducing MgO-coated CNTs does not affect the biocompatibility and corrosion resistance of AZ31, which indicates excellent application potential in the biomedical field [95].

### 2.3. Graphene

Graphene has been considered as one of the ideal reinforcements because of its outstanding mechanical properties. In addition, owing to the excellent thermal conductivity and the formation of a lubricating layer between sliding surfaces, graphene nanoplatelets (GNPs) can substantially improve the wear resistance of MMCs [96,97]. However, agglomeration and low interfacial wettability limit the enhancement effect of GNPs [98]. Many coating materials, including metal and oxide, have been investigated to solve these problems. 

In metallic coatings, Ye et al. [99] prepared a Mg-9Al composite reinforced with Ni-nanoparticle-coated graphene nanosheets, and the Ni coating enhanced the grain refinement effect and compressive properties. A semi-coherent interface of Mg_2_Ni/Mg formed, and the bonding strength was improved. In addition, continuous Ni-coated GNPs can also improve the compressive properties, wear properties, and microhardness [97]. Zhou et al. [100] demonstrated that interface strengthening, effective load transfer, and dislocation strengthening were the main strengthening mechanisms of Ni-GNPs/Mg composites through molecular dynamics simulations. Moreover, Zhao et al. [13,96,101] used the organic magnesium chemical reduction method to realize the deposition of a Mg nanoparticle coating on GNPs, and the reaction equations are shown below.

(5) $$n-C_{2}H_{5}Br+Mg\to n-C_{2}H_{5}MgBr$$

(6) $$C_{2}H_{5}MgBr+NaH\to C_{2}H_{6}+NaBr+Mg$$

**Figure 7:**
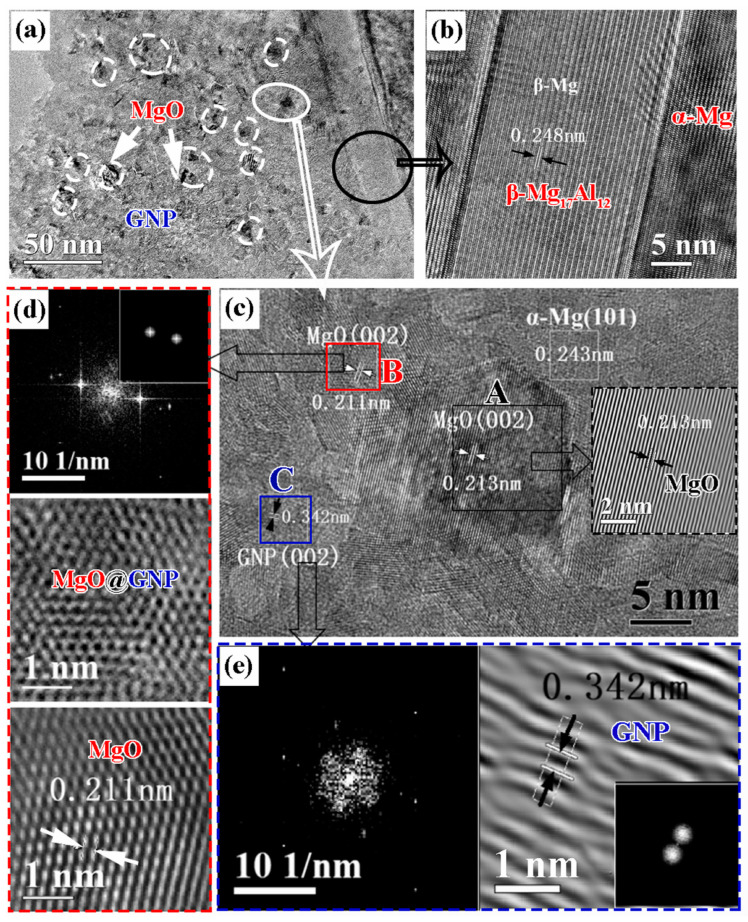
(**a**) TEM image of the composite, HRTEM image of (**b**) β-Mg_17_Al_12_ phase, and (**c**) MgO nanoparticle. SAED and IFFT images of (**d**) region B and (**e**) region C. Reproduced from [96] with permission of the publisher (Elsevier).

This method improves the interfacial wettability and makes GNPs dispersed uniformly while avoiding the introduction of other elements. As shown in Figure 7, β-Mg_17_Al_12_ phases nucleate heterogeneously, and the in situ generation of MgO, which is a sign of nano-scale contacts and tight interfacial bonding, is observed at the interface. The hardness and wear resistance are largely improved. 

In oxide coatings, Wang et al. [102] modified graphene oxide (GO) with ZnO by coprecipitation, and the interfacial wettability is improved through MgO formed in the in situ reaction. And the interfacial condition of TiO_2_-coated GO in AZ61 is similar to that of CF, which improves the mechanical properties and biological corrosion resistance [103]. Furthermore, directly using MgO as a coating material can also strengthen interfacial bonding and corrosion resistance, which expands the application of MMCs in the biomedical field [104]. Similar to CNTs, when the content of reduced graphene oxide exceeds 3 wt%, agglomeration and the accompanying inner flaws in the matrix will decrease the mechanical properties of the composites [104]. In addition, Li et al. [105] proposed a novel process of GNP-reinforced MMCs that combines the in situ CO_2_/Mg reaction to synthesize GNPs and hot extrusion. The in situ GNPs are coated with MgO, which contributes to the uniform dispersion of GNPs. Load transfer, grain refinement, and the pinning effect result in an improvement in the mechanical properties.

### 2.4. Other Carbon Reinforcements

In addition to the research above, several carbon materials have also been studied. By coating TiC or Cr on diamond, the thermal conductivity of diamond/Mg composites is significantly improved [106,107]. Olszówka-Myalska et al. [108] applied amorphous carbon with an irregular shape as the reinforcement and coated it with SiO_2_ by the sol–gel method. 

## 3. Ceramic-Reinforced MMCs

Ceramic materials have a high mechanical strength, hardness, and melting point, which are preferred in MMCs. In particular, nano-scale reinforcements can significantly improve the performance of composites because of their pinning of grain boundary movement and a fine-grain strengthening effect [109,110]. The commonly used ceramic-based reinforcements mainly include metal oxides, carbides, nitrides, etc. The morphology of ceramic reinforcements includes particles, fibers, and whiskers. Similar to carbon-based reinforcements, ceramics are also faced with problems such as poor interfacial reaction, bad wettability, and agglomeration. The following introduces several ceramic-reinforced MMCs that are improved through coating treatment. 

### 3.1. SiC Particle

**Figure 8:**
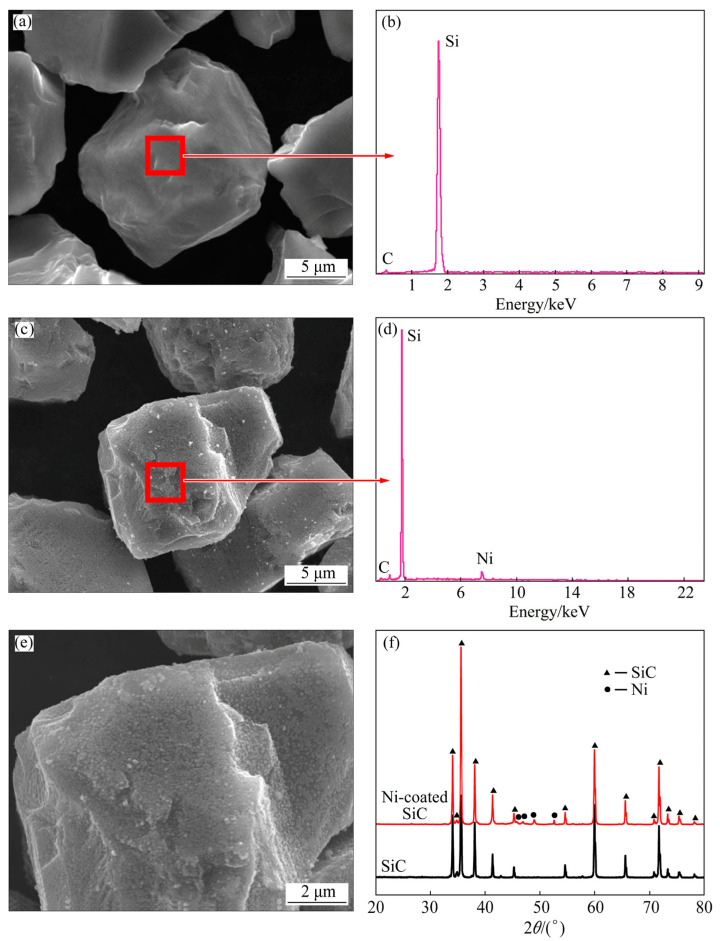
SEM micrographs of uncoated (**a**) and Ni-coated (**c**,**e**) SiC_p_ and corresponding EDS results (**b**,**d**). XRD patterns of Ni-coated and uncoated SiC_p_ (**f**). Reproduced from [111] with permission of the publisher (Elsevier).

SiC particle (SiC_p_) has the advantages of great mechanical properties, high temperature resistance, and low costs [111]. To stimulate the best performance of SiC_p_-reinforced MMCs, it is necessary to reduce the size of the particles and maintain good dispersion and interfacial bonding [112]. Coating treatment and high-temperature oxidation, as two widely used methods, can meet the above requirements. However, SiC_p_ has the problem of agglomeration at high temperatures [111]. Much research has focused on the coating treatment of SiC_p_, such as Cu [14,113], Ni [111], and Ni-P [114]. As shown in Figure 8, the change in the surface morphology and composition proves that the pure Ni coating can be plated on the surface of SiC_p_. Furthermore, coated SiC_p_ similarly has the effect of grain refinement, and the damping capacity at high temperatures is improved [14,111]. In addition to particles, coating treatment with Cu is also utilized to prevent interfacial reactions during the preparation of the Mg/SiC fiber precursor [115].

### 3.2. Al_2_O_3_ Particle

The addition of Al_2_O_3_ nano-particles can improve the strength, ductility, and hardness of MMCs [116,117,118]. In addition, the addition ofAl_2_O_3_ at an appropriate concentration can effectively change the corrosion properties to some extent [119]. Compared with optimizing the processing technology of composites, the coating treatment of Al_2_O_3_ nano-particles is a simple and effective method. Kumar et al. [120,121] prepared Ni-coated Al_2_O_3_ by electroless plating and developed an MMC with a semi-solid stir-casting technique. The strength and plasticity of the MMC are both improved. In addition, the Ni coating improves mechanical properties such as impact and fatigue response because metal-metal interactions are stronger than metal-ceramic interactions. The coating plays the role of load transfer and delaying crack initiation and propagation. In addition, Guo et al. [24] coated Al_2_O_3_ hollow spheres with MgO to prevent the interfacial reaction and investigated the thermal and mechanical properties of the composites by experiments and simulation. 

### 3.3. Whisker

Whiskers are micro-scaled or nano-scaled short fibers grown from high-purity single crystals. They have a high strength, high Young’s modulus, low density, and low coefficient of thermal expansion [122,123]. The whisker reinforcements commonly used in MMCs are magnesium borate whiskers (Mg_2_B_2_O_5_w) [124,125,126], aluminum borate whiskers (Al_18_B_4_O_33_w) [127,128,129], SiC whiskers [130], and Al_2_O_3_ whiskers [21]. Among them, SiC whiskers have the best chemical stability and mechanical properties, but the cost is more than ten times that of borate whiskers [122,129,130]. Borate whiskers, as a cost-effective alternative, also have good mechanical properties. However, the interfacial reactions of borate whiskers and the Mg matrix will affect the properties of MMCs. In an AZ91 matrix, a uniform and dense interfacial reaction layer with the composition of MgO [129] or MgAl_2_O_4_ [131] forms on Al_18_B_4_O_33_w, which acts as a barrier to prevent further reaction and improve stability. A similar MgO reaction layer also exists in Al_18_B_4_O_33_w/MB8 and Al_18_B_4_O_33_w/MB15 composites [122]. On the contrary, interfacial reaction will damage the whiskers in Al_18_B_4_O_33_w/ZK60 composites [128]. For the Mg_2_B_2_O_5_w/AZ91 composites, the interfacial reaction layer is uneven and rough [132] or does not exist [125]. The bad interfacial reaction above can be improved through coatings the whiskers.

**Figure 9:**
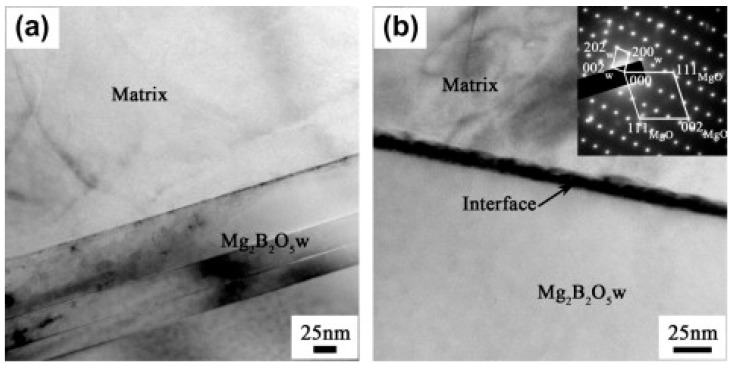
TEM micrographs of the interfaces in the composites: (**a**) Mg_2_B_2_O_5_w/AZ91D and (**b**) Mg_2_B_2_O_5_w/TiO_2_/AZ91D. Reproduced from [125] with permission of the publisher (Elsevier).

Borate whiskers are commonly optimized by surface coatings, while Al_2_O_3_ whiskers and SiC whiskers are modified through other surface treatments [21,130]. Because Al_18_B_4_O_33_w can react with magnesium to form a dense reaction layer, Sasaki et al. [128] evaporated pure Mg on the whiskers and made them react to produce a MgAl_2_O_4_ layer before adding it into the ZK60 matrix. The layer protects the whiskers and gives the composites a higher hardness. Li et al. [125] reported a synthesis of TiO_2_-coated Mg_2_B_2_O_5_w via the sol–gel method, and the flexural strength and flexural modulus of its AZ91D-based composite is 40% and 35% higher than that of the Mg_2_B_2_O_5_w/AZ91D composite, respectively. By coating borate whiskers with CuO [124] or ZnO [126], the tensile strength and elongation to fracture of the MMCs are higher than composites with uncoated whiskers. As shown in Figure 9, these oxide matrix coatings react with the Mg matrix to form a protective layer and enhance interfacial bonding. In addition, Mo et al. [127] prepared BN-coated Al_18_B_4_O_33_w via the sol–gel method and post-thermal-treatment methods, and the coating can effectively protect the whiskers from interfacial reaction. The hardness of AZ91D-based composites is remarkably increased to 114 HV with a suitable whisker content. 

### 3.4. Hydroxyapatite

**Figure 10:**
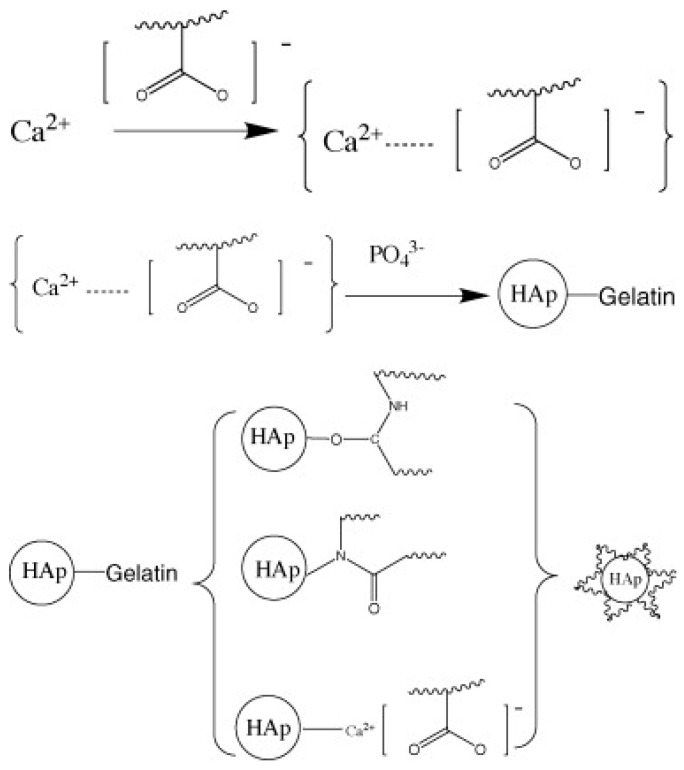
Proposed reaction routes for coating gelatin on HAp nanorods. Reproduced from [135] with permission of the publisher (Elsevier).

Mg and its alloys have broad prospects as implant materials because of their biodegradability and mechanical properties matching human bones [133]. However, its poor corrosion resistance limits its applications. The addition of a strengthening phase can refine the grain, which makes MMCs one of the research hotspots. Hydroxyapatite (HA), as a commonly used reinforcement in biomedical MMCs, has good biocompatibility, low solubility in our body fluid, and similar structures to natural bone [134]. As shown in Figure 10, Chen et al. [135] prepared gelatin-coated HA nanorods using a direct chemical reaction, which improves the dispersibility and stability without a loss of biocompatibility. Researchers have added nanorods with 1 wt% in Mg-Zn-Zr, and the mechanical properties of the composite were marginally enhanced, while the corrosion resistance and biocompatibility exhibited significant improvements [136,137,138]. Furthermore, applying MgO as a coating material can also enhance the corrosion resistance [139]. The same coating method has also been applied to tricalcium phosphate ceramic nanoparticles (TCP) with good results [140]. The possible mechanism of HA/Mg-Zn-Zr and MgO-HA/Mg-Zn-Zr in a simulated body fluid is shown below. At the early stage of immersion, with the increase in the corrosion time, the Mg(OH)_2_ layer and the Ca-P particle layer on the surface of MgO-HA/ Mg-Zn-Zr grow alternately and compete with each other. The two types of layers grow opposite and cover each other so that the corrosion develops evenly and basically inhibits the pitting process. On the contrary, the HA/Mg-Zn-Zr composite has a poorer corrosion resistance because of HA agglomeration, which prevents the formation of a dense and continuous protective reaction layer. Additionally, Radha et al. [29] coated metallic Sn on HA particles by electroless plating, which helps in the refinement of grains and the uniform distribution of HA particles. Meanwhile, the coating reduces the biodegradation of MMCs by preventing the dissociation of HA and the formation of detrimental phases such as CaSn. 

### 3.5. Fly Ash Cenospheres

**Figure 11:**
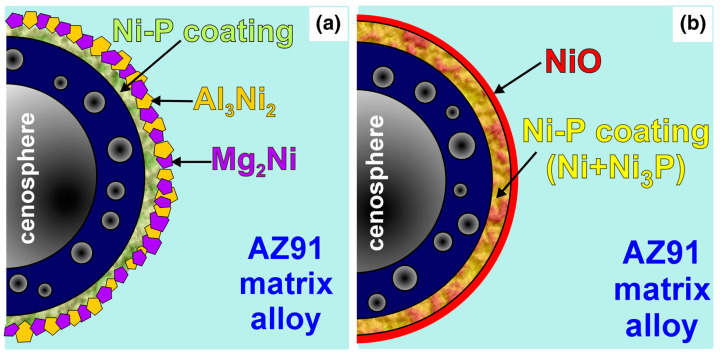
Schemes of interfaces between components in investigated AZ91/FAs (**a**) and AZ91/FAs (heat-treated) (**b**) composites. Reproduced from [150] with permission of the publisher (Springer Link).

Fly ash cenospheres are a new type of multifunctional particle material extracted from fly ash discharged from coal-fired power plants and an important by-product of the advanced utilization of fly ash. They are hollow, thin-walled ceramic spheres with a size of 5–500 μm and a density of 0.4–0.7 g/cm^3^ [141,142,143]. Cenosphere-reinforced metal matrix composites are also called syntactic foams, and the addition of cenospheres improves the compressive properties and wear performance of MMCs [144,145,146]. The main problems that limit the properties of these composites are mechanical damage and chemical reactions with the liquid Mg alloy matrix during the fabrication process [147,148,149]. Through the Ni-P coating treatment by electroless plating, the stability and dispersion of cenopheres are substantially improved. Braszczyńska-Malik et al. [149,150,151] successfully fabricated AZ91 matrix composites with Ni-P-coated fly ash cenospheres by the negative pressure infiltration technique. The Ni-P coating reacted with the molten alloy matrix to form Al_3_Ni_2_ and Mg_2_Ni intermetallic phases at the interfaces. A NiO external layer and internal reactions of the coating are formed during the heat treatment. As shown in Figure 11, both interfaces stably and effectively protect the cenospheres from the reactions and mechanical damage. 

## 4. Discussion

### 4.1. The Effect of Coatings

By summarizing the above research, we can conclude that coatings applied to reinforcements can improve the dispersion, enhance interfacial bonding and wettability, inhibit bad interfacial reactions and corrosion, retard element diffusion, increase thermal stability, relieve interfacial stress, transfer load and so on. After introducing coatings on the interfaces between reinforcements and the alloy matrix, the comprehensive properties of MMCs are enhanced. Furthermore, some studies have proven that MgO coatings have a positive effect on reducing the corrosion rate, and this phenomenon has been found in MMCs reinforced with HA, CNT, and reduced graphene oxide [95,104,139]. Moreover, many oxide coatings can react with the matrix to form MgO. We consider that this can also improve the corrosion resistance of MMCs to a certain extent.

However, we found a phenomenon that there are a larger number of research articles dedicated to SiC, Al_2_O_3_, and TiC reinforcements, while the coated reinforcements with a larger number of research articles are CF and CNT. We consider the reasons for this as follows. Firstly, carbon may react with elements in the alloy matrix. Therefore, carbon materials need more protection. Secondly, the interface of the particle reinforcements, especially nanoparticles, is simpler than with fibers, nanotubes, and whiskers, while fibers and nanotubes have bigger continuous interfaces that are easier to coat. Finally, the agglomeration problem of nanoparticles can also not be solved well by coating treatment. It needs to be solved in combination with an improvement in the manufacturing process. The commonly used dispersion method is ball-milling, and some novel techniques such as semi-solid stir-casting are adopted [117].

Among all the coating materials, metals and oxides are most used on different reinforcements. This may be due to their excellent performance, reasonable cost, and mature preparation methods. In order to compare the effects of different coatings and reinforcements, the tensile strength data are listed in Table 1. It should be noted that the effect of different coatings and reinforcements on MMCs is reflected in different aspects, such as wear resistance, corrosion resistance, damping properties, etc. This review only makes the comparison from the perspective of tensile strength. In general, the tensile strength of MMCs is improved more or less through coating treatments on the surface of reinforcements. The results show that CF and whiskers have a much larger fraction in MMCs than other reinforcements, and the average strengthening effect is also higher. The highest tensile strength is 1080 MPa shown in ref. [51]. The primary reason why other reinforcements struggle to increase the fractions is agglomeration. Even if a coating is introduced, the agglomeration problem still exists at higher concentrations [86]. The tensile strength of some uncoated systems is lower than that of the corresponding matrix. This shows that the coating solves the problems of agglomeration and undesirable interfacial reaction to some extent.

### 4.2. Future Recommendations

Coating treatment is an important method used to improve the interfaces between reinforcements and Mg matrices. Based on the existing research, we consider that the future research direction is as follows:

1. New types of coatings and reinforcements need to be explored. This can be studied from the angle of economy or high performance. In addition, new applications of coatings at the micro-scale and nano-scale can be developed, such as preventing the oxidation of the metal powder during processing [119].
2. Furthermore, more detailed and nano-scale studies of the interface structure are needed, and precise structure-function relationships need to be established. The interfaces between the reinforcements and coatings and the interfaces between the coatings and the matrix both need to be further investigated.
3. The durability of coatings needs to be further studied. Due to the activity and erodibility of Mg, it is critical to make the strengthening effect of the composites and coatings last longer. Whether there is coating degradation and its impact on the properties of composites are worthy of study.
4. In addition to coating treatments, there are many surface modification methods for reinforcements, such as surface grafting [12,152]. There are few studies on the comparison between different coatings, different reinforcements, and different surface modification methods. Only by making sufficient comparisons can we give practical suggestions for future application selection.

## 5. Conclusions

In summary, different types of coatings on the reinforcements in MMCs were reviewed in detail in this paper. Coating materials include metals, oxides, PyC, BN, gelatin, etc., and the preparation methods include the sol–gel method, CVD, electroless plating, etc. The main roles of coatings on the reinforcements are improving dispersion, enhancing interfacial bonding and wettability, inhibiting bad interfacial reactions and corrosion, retarding element diffusion, increasing thermal stability, relieving interfacial stress, and transferring load. Through introducing coatings on the reinforcements, the mechanical properties, wear resistance, and other properties can be drastically improved.

## Figures and Tables

**Table 1:** Tensile strength data of different MMCs.

Reference	Fraction of Reinforcements	Matrix	Reinforcement	Coatings	Tensile Strength of Matrix (MPa)	Tensile Strength of Uncoated Reinforcement/Matrix (MPa)	Tensile Strength of Coated Reinforcement/Matrix (MPa)
[30]	-	AE44	CF	PyC	185	-	412
[35]	45 vol%	AZ31	CF	PyC	-	256	426
[35]	45 vol%	AZ91	CF	PyC	-	227	401
[60]	45 vol%	AZ91D	CF	PyC	-	308	416
[59]	-	AZ91D	CF	PyC	180	220	400
[63]	45 vol%	AZ91D	CF	PyC	180	235	400
[63]	45 vol%	AZ91D	CF	TiO_2_	180	235	333
[57]	10 vol%	AZ91	CF	TiO_2_	153	-	306
[49]	45 vol%	Mg	CF	TiO_2_	30	-	980
[49]	45 vol%	Mg	CF	Al_2_O_3_	30	-	550
[51]	45 vol%	Mg	CF	ZrO_2_	30	-	1080
[69]	0.5 wt%	Mg-Al-Ca	CF	Ni	141	-	161.8
[68]	4	Mg	CF	Ni	90	-	167
[67]	5.5 vol%	Mg	CF	Ni	213.78	187.35	241.82
[72]	7.5 wt%	AZ91D	CF	Si	336.8	-	470
[93]	3 wt%	AZ91	CNT	MgO	215	301	331
[92]	3 wt%	AZ91	CNT	MgO	168	250	284
[91]	1 wt%	AZ91-Pr	CNT	TiO_2_	261.32	258.47	389.67
[94]	1 wt%	Mg-0.4Zn	CNT	NiO	159	176	212
[77]	2 wt%	Mg	CNT	Ni	-	205.9	304.5
[78]	1 wt%	AZ91D	CNT	Ni	321	-	382
[79]	0.3 wt%	Mg	CNT	Ni	171	163	237
[87]	0.3 wt%	Mg	CNT	Al	153	-	227
[105]	0.35 vol%	Mg-6Zn	Graphene	MgO	235.71	-	330
[102]	0.3 vol%	Mg-6Zn	Graphene	ZnO	269	-	316
[140]	1 wt%	Mg-Zn3-0.8Zr	HA	MgO	306	317.53	346.11
[139]	1 wt%	Mg-Zn3-0.8Zr	HA	MgO	306	320	325
[138]	1 wt%	Mg-3Zn-0.5Zr	HA	Gelatin	-	275	285
[137]	1 wt%	Mg-Zn3-0.8Zr	HA	Gelatin	-	315	325
[111]	9 vol%	AZ61	SiC particle	Ni	272.8	320	336
[121]	2 wt%	AZ91E	Al_2_O_3_ particle	Ni	162.77	-	221.09
[124]	36 vol%	AZ31B	Mg_2_B_2_O_5_ whisker	CuO	-	181	249
[126]	36 vol%	AZ31B	Mg_2_B_2_O_5_ whisker	ZnO	-	181	305

## Data Availability

No new data were created or analyzed in this study.
